# The study of factors associated with pregnancy outcomes in patients with systemic lupus erythematosus

**DOI:** 10.1186/s13104-020-05039-9

**Published:** 2020-03-30

**Authors:** Batool Zamani, Mohammad Shayestehpour, Farifteh Esfahanian, Hossein Akbari

**Affiliations:** 1 Autoimmune Diseases Research Center, Kashan University of Medical Sciences, Kashan, Iran; 2 Department of Microbiology and Immunology, Faculty of Medicine, Kashan University of Medical Sciences, Kashan, Iran; 3 Department of Biostatistics and Public Health, Faculty of Health, Kashan University of Medical Sciences, Pezeshk Blvd, 5th of Qotb-e Ravandi Blvd, P.O.Box: 8715973449, Kashan, Iran

**Keywords:** Abortion, Pre-eclampsia, Pregnancy outcome, Stillbirth, Systemic lupus erythematosus

## Abstract

**Objectives:**

Systemic lupus erythematosus (SLE) is an autoimmune disease that can lead to unfavorable pregnancy complications in women. This study aimed to evaluate the factors associated with pregnancy outcomes in patients with SLE.

**Results:**

Fifty-nine pregnant women with SLE (121 pregnancies) participated in this retrospective cohort study. The mean age of the patients was 33.74 ± 3.80 years (range 21 to 48 years). Fetal loss occurred in 43.8% of pregnancies. The most common laboratory findings in SLE patients were antinuclear antibody (81.4%) and anti-ds DNA positivity (54.2%). High levels of C-reactive protein (CRP) during pregnancy, renal involvement, anti-double-stranded DNA positivity, anti-phospholipid antibody (APA) positivity and younger age at disease onset were significantly correlated with unfavourable pregnancy outcomes. A significant difference was observed between duration of SLE and low birth weight (*P *= 0.003), pre-eclampsia (*P *= 0.012) and still birth (*P *= 0.036). High CRP, APA positivity, anti-dsDNA positivity and kidney involvement were predictors of adverse pregnancy outcomes in SLE patients. Renal involvement increased risk of pregnancy with complication 8.5 times (OR = 8.5, 95% CI 1.396–63.373, *P *= 0.017). Antiphospholipid syndrome (APS) also was associated with an odds ratio of 5.18 (95% CI 1.681–13.647, *P *= 0.001).

## Introduction

Systemic lupus erythematosus (SLE) is an autoimmune disease with an annual incidence of 4–35 cases per 100,000 people, which predominantly find in females of child-bearing age [1]. Disease relapse can occur in throughout the entire pregnancy and often immediately after termination of pregnancy. Several studies reported the SLE relapse during pregnancy and considered an important role for estrogen in causing disease [2]. Although the majority of studies showed an increased SLE relapse during pregnancy, but some believe that the frequency of flares during pregnancy is similar to non-pregnancy [2–4]. In a study conducted by Urowits et al., disease activity at the onset of pregnancy did not predict the risk of exacerbation during pregnancy. They reported that patients with inactive SLE less likely to experience a flare of disease [5]. However, methodological differences and the use of different sample sizes may be the cause of discrepancies in the obtained results. A lupus flare during pregnancy is dependent on disease’s activity at the time of fertilization. The frequency of flare has been reported 7–33% in women who were in remission for at least 6 months before conception, while 61–67% of patients with active SLE at the onset of pregnancy have been a disease flare. However, there is no consensus among researchers that increased lupus activity is due to pregnancy or spontaneous fluctuation of disease [6–8].

Some pregnancy adverse outcomes can be related to SLE, including pre-eclampsia, abortion, preterm birth, stillbirth, and fetal growth restriction. A recent meta-analysis study showed that the SLE had a high impact on maternal and fetal outcomes following pregnancy. Pre-eclampsia and hypertension had significantly higher rates among women with SLE (relative risk (RR): 1.85 1.91, 95% confidence interval (CI) 1.44–2.53; P = 0.00001) and (RR: 1.99, 95% CI 1.54–2.56; P = 0.00001) respectively. In addition, thromboembolic disease and abortion were also significantly higher in the SLE patients [9]. Nevertheless, several studies have shown SLE to have unfavorable pregnancy outcomes, the factors associated with such complications seem to be varied from region to region. There are limited studies conducted on pregnancy outcomes among Iranian women with SLE. Considering the high prevalence of SLE in women and importance of maternal–fetal health, this study aimed to evaluate factors associated with pregnancy outcome in patients with systemic lupus erythematosus.

## Main text

### Methods and materials

Total pregnant women with SLE (59 patients, 121 pregnancies) referred to the rheumatology clinic at the Shahid Beheshti Hospital of Kashan University of Medical Sciences in Kashan City between 2003 and 2017 were participated in this retrospective cohort study. Study population included pregnant women with pre-existing SLE and new onset SLE. All SLE patients had the American college of rheumatology diagnostic criteria. Disease activity was estimated based on the systemic lupus erythematosus disease activity index (SLEDAI). Patients were categorized into three groups, according to SLEDAI score: remission (6>), mild to moderate activity [6–12] and high activity (>12). The medical records of patients were comprehensively reviewed to collect demographic, clinical and laboratory data. Recorded findings included age, duration of illness, disease activity, blood pressure, drugs, number of pregnancies, abortion and intrauterine fetal death. Patients with incomplete medical records were excluded from the study. Laboratory parameters included complete blood cell (CBC), erythrocyte sedimentation rate (ESR), C-reactive protein (CRP), and venereal disease research laboratory (VDRL). Immunologic parameters, including total complement activity (CH50: 101–300) anti-double-stranded DNA antibodies (anti-dsDNA: < 70 IU/mL), antiphospholipid antibodies (APA: < 10 IU/mL), complement 3 (90–180 mg/dL), and complement 4 (13–75 mg/dL) were evaluated by enzyme-linked immunosorbent assay (ELISA). Antinuclear antibodies (ANA: < 1:80) were measured by indirect immunofluorescence assay (IFA). All patients were followed up during pregnancy by a gynecologist, nephrologist and rheumatologist. Maternal complications, including PROM (rupture of membranes prior to the onset of labor at or beyond 37 weeks’ gestation), Pre-eclampsia (systolic blood pressure ≥ 140 mmHg and/or diastolic blood pressure (DBP) ≥ 90 mmHg after 20 weeks of gestation in a previously normotensive woman). In addition, outcomes related to fetal, including LBW (a birth weight of less than 2500 g), stillbirth (fetal death ≥ 20 weeks of gestation), abortion (pregnancy termination before 20 weeks’ gestation or with a fetus born weighing < 500 g, and Preterm labor (birth before 37 weeks of pregnancy) were checked by a pediatrician.

#### Statistical analysis

The statistical analysis was conducted using the Statistical Package for Social Sciences (SPSS) version 21.0 (SPSS Inc, Chicago, IL, USA). Qualitative data were analyzed by Chi-square test and Fisher’s exact test. The continuous variables were analyzed by using Student’s t test. Variables with a P ≤ 0.10 in these analyses were entered into a multiple logistic regression model to determine the predictive factors for adverse pregnancy outcomes with adjustment of confounding factors. Adjusted odds ratios (OR) are presented at 95% confidence intervals (CI). In all tests, *P* values less than 0.05 were considered to be statistically significant.

### Results

In this study, 121 pregnancies in 59 SLE patients were evaluated between 2003 and 2017. The mean age of the patients was 33.74 ± 3.80 years ranging from 21 to 48 years old. A total of 96 pregnancies (79.3%) were linked to adverse outcomes. Abortion and stillbirth occurred in 47 (38.8%) and 6 (5%) pregnancies, respectively. Thirty-eight of pregnancies (56.2%) led to a live birth. The most abundant laboratory findings in SLE patients were antinuclear antibody (81.4%) and Anti-ds DNA positivity (54.2%). Six patients had a history of lupus nephritis (three cases with class IV nephritis, two cases with class II, and one case with class III) Renal involvement (10.2%) and CRP test before pregnancy (11.9%) was less common. Pregnancy occurred in 117 cases (97%) in the quiescent phase of SLE, and in cases with active diseases (3%). Twenty-three women had a relapse of SLE (19%). Fifty-seven pregnant women (97%) had pre-existing lupus, while two women (3%) referred with SLE onset in pregnancy. Patients during pregnancy received 5–10 mg Prednisolone and 200 mg Hydroxychloroquine daily. Frequency of pregnancy complications according to laboratory findings in pregnant women with SLE is summarized in Table 1. There was a significant correlation between ANA positivity and abortion (*P *= 0.016) or preterm birth (*P *= 0.002). Increasing ESR, high CRP during pregnancy and renal involvement was significantly associated with stillbirth. Occurrence of poor outcomes, including PROM, LBW, pre-eclampsia, preterm labor and stillbirth was correlated with maternal anti-dsDNA positivity. Antiphospholipid antibodies (APA) were detected in 46 patients (38%). Presence of APA antibody in pregnant women was significantly linked with abortion (*P *= 0.017).

**Table 1:** Frequency of pregnancy complications according to laboratory findings in pregnant women with SLE

Laboratory findings	Outcome (number: frequency)													
	PROM	P value	LBW	P value	Pre-eclampsia	P value	Neonatal lupus	P value	Preterm labor	P value	Abortion	P value	Still birth	P value
***ESR***														
Low	1	0.208	3	0.108	3	0.014	1	0.413	4	0.001	23	0.175	0	0.041
High	5		11		16		0		27		24		1	
***CRP***														
Low	2	0.005	10	NS	12	0.356	1	NS	18	0.047	37	0.183	2	0.005
High	4		4		7		0		13		10		4	
***ANA***														
Negative	0	0.594	2	NS	2	0.305	0	NS	0	0.002	14	0.016	0	0.224
Positive	6		12		17		1		31		33		6	
***Renal involvement***														
Negative	6	0.059	10	0.221	10	0.001	1	NS	24	0.239	41	0794	3	0.042
Positive	0		14		9		0		7		6		3	
***Anti-ds DNA***														
Negative	5	0.008	2	0.025	14	0.043	1	0.421	18	0.005	23	0.228	0	0.039
Positive	1		12		15		0		13		24		6	
***APS***														
Negative	6	0.095	12	0.136	14	0.448	1	NS	18	0.272	25	0.017	2	0.178
Positive	0		2		5		0		13		22		4	
***Anemia***														
Negative	2	0.043	10	0.222	13	0.242	0	0.438	10	0.002	23	0.199	5	0.229
Positive	4		4		6		1		21		24		1	

*PROM* premature rupture of membranes, *LBW* low birth weight, *ESR* erythrocyte sedimentation rate, *CRP*: C-reactive protein, *ANA* antinuclear antibody, *Anti-ds DNA* anti-double stranded DNA, *APS* antiphospholipid syndrome

Pregnancy in 4.3% of women who had a duration of SLE less than 5 years led to LBW. This complication in patients with a duration ≥ 6 years was 21.6%. A significant difference was observed between SLE duration and LBW (*P *= 0.003), pre-eclampsia (*P *= 0.012), stillbirth (*P *= 0.036) (Table 2).

**Table 2:** Pregnancy outcomes according to disease duration in SLE patients

Outcome	Duration of SLE		
	1–5 year	≥ 6 year	*P* value
PROM	3 (4/3)	3 (5/9)	0.69
LBW	3 (4/3)	11 (21/6)	0.003
Pre-eclampsia	6 (8/6)	13 (25/5)	0.012
Neonatal lupus	1 (1/4)	0	NS
Preterm labor	19 (27/1)	12 (23/5)	0.653
Abortion	30 (42/9)	17 (33/3)	0.289
Still birth	1 (1/4)	5 (8/9)	0.036

Logistic regression analysis was performed to assess the association of laboratory findings and adverse pregnancy outcomes (Table 3). High CRP during pregnancy, kidney involvement, anti ds-DNA positivity, APA positivity and younger ages at disease onset was significantly correlated with severe pregnancy complications. Renal involvement was the most important factor associated with pregnancy outcomes and increased the risk of complicated pregnancy 8.5 times (OR = 8.545, CI 1.396–63.373, *P *= 0.017). Antiphospholipid syndrome (APS) also was associated with an odds ratio of 5.18 (95% CI 1.681–13.647, *P *= 0.001). Pregnancy complications were higher in younger SLE patients. Increasing every 1 year in age of disease onset decreased 93% of risk of a pregnancy with complication (OR: 0.932, 95% CI 0.865–1.009).

**Table 3:** Association of predictors with the occurrence of adverse fetal outcomes in SLE patients by multivariable logistic regression analyses

Predictors	OR (95% CI)	*P* value
Duration of disease	0.937 (0.217–1.529)	0.229
ESR during pregnancy	0.980 (0.953–1.003)	0.096
CRP during pregnancy	2.896 (0.962–8.674)	0.037
Renal involvement	8.545 (1.396–63.373)	0.017
Anti-ds DNA	0.219 (0.079–0.655)	0.002
APS	5.181 (1.681–13.647)	0.001
Anemia	1.570 (0.676–3.985)	0.320
Age at onset SLE	0.932 (0.865–1.009)	0.018

### Discussion

Despite the recent progress in management of pregnant women with SLE, results of the present study show that SLE during pregnancy is still associated with the incidence of adverse pregnancy outcomes. One hundred twenty-one pregnancies were occurred in 59 women with SLE. Spontaneous abortion, preterm labor, SLE relapse and stillbirth were observed in 38.8%, 25.6%, 19% and 5% of pregnancies, respectively.

In this study, fetal loss of 43.8% was observed, which is high compared to other reports. In the previous studies, abortion has been one of the most common pregnancy outcomes in women with SLE. This complication in study of Yuen et al. [10] and Parastandechehr et al. [11] were reported 19.5% and 19%, respectively. Clowse and colleagues were founded that the activity of SLE during pregnancy led to a threefold increase in spontaneous abortion [12]. Fetal loss, including abortion and stillbirth in the study of Park et al. was 17.7% [13]. In the present study, abortion incidence is higher than the above-mentioned researches) 38.8%), because many women with frequent abortion were referred to Kashan’s rheumatology clinic to find the cause of their fetal loss. Diagnosis of lupus in these women is a possible reason for increasing the incidence of abortion in our study.

In the current study, live-birth rate in SLE patients was 56.2%, which is less than the 80.5–91.9% reported in other studies. Preterm birth was occurred in 25.8% of pregnancies. Many studies have reported a high rate of preterm birth as an adverse pregnancy outcome in SLE patients. The frequency of this complication in studies conducted by Limba et al. [14], Smyth et al. [4], Park et al. [13] and Megan et al. [12] were 43%, 39%, 25.5% and 23.7%, respectively. In this study, pre-eclampsia observed in 15.7% of patients. Previous similar studies have reported a wide range of pre-eclampsia in pregnant women with SLE from 3 to 26%. The frequency of SLE relapse during pregnancy in our study population was 19%. The previous studies have been reported the relapse rates ranged from 13 to 68%. Generally, the frequency of outcomes evaluated in this study is within the reported range from other studies [15–17].

In this study, the increasing SLE duration is significantly raised the incidence of some pregnancy complications, including LBW (*P *= 0.003), pre-eclampsia (*P *= 0.012) and stillbirth (*P *= 0.036). Impact of disease duration on pregnancy outcomes has not been evaluated in the previous studies.

The results of the present study demonstrate that high levels of CRP, APS positivity, anti-dsDNA positivity and renal involvement are predictors of adverse pregnancy outcomes in SLE patients. These data are in consistent with previous findings [16, 18–20]. The age of SLE onset was impressed the pregnancy complications in our assessment in contrast with the report of Feld et al. [19].

The impact of SLE renal disease on pregnancy outcomes is debatable. Several previous studies showed that lupus nephritis was significantly linked with severe pregnancy outcomes. By contrast, some studies have not indicated such relationship [16, 17]. This difference may have been created by the number of patients in various studies. In addition, study design, population under study, treatment or lupus nephritis status may explain the different results obtained. Based on our findings, renal involvement is an important risk factor for adverse pregnancy outcomes, including pre-eclampsia (*P *= 0.017) and still birth (*P *= 0.042). SLE patients with renal damage were 8.5 time (OR = 8.545, CI 1.396–63.373, *P *= 0.017) more likely to have a severe pregnancy outcome. There is a significant association between APS and poor pregnancy outcomes (*P *= 0.001), especially abortion (*P *= 0.017), that was similar to several studies [9, 13, 21]. This conferred approximately a 5.2-fold increased risk of unfavorable outcomes (OR: 5.18, 95% CI 1.681–13.647, *P *= 0.001) when compared to APS-negative patients. Fled et al. also have reported that APS is associated with an odds ratio of 5.31 [19].

In conclusion, Frequency of pregnancy complications was high in women with pre-existing SLE. The most unfavorable pregnancy outcome in women with SLE was spontaneous abortion. Duration of SLE prior to pregnancy was significantly associated with some outcomes, including LBW, pre-eclampsia, and still birth. High CRP, renal involvement, anti-dsDNA positivity, APS positivity and low age at the onset of the disease increased the risk of pregnancy complications.

## Limitations

The study limitations were its retrospective nature and small sample size. Some pregnant women with SLE (24 cases) were excluded due to incomplete data in their medical records. This study was limited by the amount of information obtained from medical records. We could not access data on some variables.

## Data Availability

No additional file is available for this study; all the data are included in the manuscript.

## Abbreviations

SLE: Systemic lupus erythematosus
APA: Antiphospholipid syndrome
APS: Anti-phospholipid antibody
CRP: C-reactive protein
PROM: Premature rupture of membranes
LBW: Low birth weight
ESR: Erythrocyte sedimentation rate

## References

[1] Draborg AH, Duus K, Houen G (2012). Epstein–Barr virus and systemic lupus erythematosus. Clin Dev Immunol.

[2] Dhar JP, Essenmacher LM, Ager JW, Sokol RJ (2005). Pregnancy outcomes before and after a diagnosis of systemic lupus erythematosus. Am J Obstet Gynecol.

[3] Clowse ME (2007). Lupus activity in pregnancy. Rheum Dis Clin N Am.

[4] Smyth A, Oliveira GH, Lahr BD, Bailey KR, Norby SM, Garovic VD (2010). A systematic review and meta-analysis of pregnancy outcomes in patients with systemic lupus erythematosus and lupus nephritis. Clin J Am Soc Nephrol.

[5] Urowitz MB, Gladman DD, Farewell VT, Stewart J, McDonald J (1993). Lupus and pregnancy studies. Arthritis Rheum.

[6] Andrade R, Sanchez ML, Alarcon GS, Fessler BJ, Fernandez M, Bertoli AM (2008). Adverse pregnancy outcomes in women with systemic lupus erythematosus from a multiethnic US cohort: LUMINA (LVI) [corrected]. Clin Exp Rheumatol.

[7] Nili F, McLeod L, O’Connell C, Sutton E, McMillan D (2013). Maternal and neonatal outcomes in pregnancies complicated by systemic lupus erythematosus: a population-based study. J Obstet Gynaecol Can.

[8] Cortés-Hernández J, Ordi-Ros J, Paredes F, Casellas M, Castillo F, Vilardell-Tarres M (2002). Clinical predictors of fetal and maternal outcome in systemic lupus erythematosus: a prospective study of 103 pregnancies. Rheumatology.

[9] Bundhun PK, Soogund MZ, Huang F (2017). Impact of systemic lupus erythematosus on maternal and fetal outcomes following pregnancy: a meta-analysis of studies published between years 2001–2016. J Autoimmun.

[10] Yan Yuen S, Krizova A, Ouimet JM, Pope JE (2008). Pregnancy outcome in systemic lupus erythematosus (sle) is improving: results from a case control study and literature review. Open Rheumatol J.

[11] Parastandechehr G, Faezi ST, Paragomi P, Akhlaghi M, Akbarian M (2016). Can pregnancy induce relapse in systemic lupus erythematosus (SLE)?. Rheumatol Res.

[12] Clowse MEB, Wallace DJ, Weisman M, James A, Criscione-Schreiber LG, Pisetsky DS (2013). Predictors of preterm birth in patients with mild systemic lupus erythematosus. Ann Rheum Dis.

[13] Park EJ, Jung H, Hwang J, Kim H, Lee J, Ahn JK (2014). Pregnancy outcomes in patients with systemic lupus erythematosus: a retrospective review of 62 pregnancies at a single tertiary center in South Korea. Int J Rheum Dis.

[14] Lima F, Buchanan NM, Khamashta MA, Kerslake S, Hughes GR (1995). Obstetric outcome in systemic lupus erythematosus. Semin Arthritis Rheum.

[15] Wu J, Ma J, Bao C, Di W, Zhang WH (2018). Pregnancy outcomes among Chinese women with and without systemic lupus erythematosus: a retrospective cohort study. BMJ Open.

[16] Phansenee S, Sekararithi R, Jatavan P, Tongsong T (2018). Pregnancy outcomes among women with systemic lupus erythematosus: a retrospective cohort study from Thailand. Lupus.

[17] Ling N, Lawson E, von Scheven E (2018). Adverse pregnancy outcomes in adolescents and young women with systemic lupus erythematosus: a national estimate. Pediatr Rheumatol Online J.

[18] Moroni G, Doria A, Giglio E, Imbasciati E, Tani C, Zen M (2016). Maternal outcome in pregnant women with lupus nephritis. A prospective multicenter study. J Autoimmun.

[19] Feld J, Kibari A, Rozenbaum M, Riskin-Mashiah S, Eder L, Laor A (2015). The fetal outcomes of pregnancies of systemic lupus erythematosus patients in northern Israel. J Maternal-Fetal Neonatal Med.

[20] Chakravarty EF, Colon I, Langen ES, Nix DA, El-Sayed YY, Genovese MC (2005). Factors that predict prematurity and preeclampsia in pregnancies that are complicated by systemic lupus erythematosus. Am J Obstet Gynecol.

[21] Karimi FZ, Saeidi M, Mirteimouri M, Maleki- Saghooni N (2017). Maternal, fetal and neonatal outcomes in pregnant women with systemic lupus erythematosus: a comprehensive review study. Int J Pediatr.

